# Transcranial sonography depicts a larger substantia nigra echogenic area in renal transplant patients on calcineurin inhibitors than on rapamycin

**DOI:** 10.1186/s12882-022-02741-7

**Published:** 2022-03-17

**Authors:** Nordeval Cavalcante Araújo, José Hermógenes Rocco Suassuna, Rita de Cássia Leite Fernandes

**Affiliations:** 1 Division of Nephrology, University of the State of Rio de Janeiro, Boulevard 28 de Setembro, 77 – Vila Isabel, Rio de Janeiro-RJ, 20551-030 Brazil; 2 Division of Neurology, Federal University of Rio de Janeiro, Rio de Janeiro, Brazil

**Keywords:** Kidney transplant, Substantia nigra echogenic area, Third ventricle width, Transcranial sonography, Calcineurin inhibitors, Rapamycin

## Abstract

**Background:**

After kidney transplantation neurologic manifestations may develop, including Parkinson’s disease (PD). An enlarged substantia nigra (SN) by transcranial sonography has been recognized as a marker of PD.

**Methods:**

In renal transplant recipients (RTRs = 95) and controls (*n* = 20), measurement of mesencephalon, SN, third ventricle, spleen and carotid intima-media thickness (cIMT) and middle cerebral artery (MCA), kidney and spleen arteries Doppler resistive index (RI) were performed.

**Results:**

RTRs had larger SN, third ventricle and cIMT and higher renal RI than controls. The SN was larger in the CNIs group than in controls and rapamycin group, while the third ventricle was similar between patients but larger than in controls. In RTRs, SN showed a direct linear correlation with spleen and the third ventricle with age, cIMT and RI of the MCA, kidney and spleen. In CNIs group the SN correlated positively with age and cIMT, while the third ventricle reproduced RTRs correlations. Rapamycin group showed a direct linear relationship between the third ventricle and age and RI of the MCA, kidney and spleen; SN showed no correlations.

**Conclusion:**

RTRs on CNIs present a larger SN area than on rapamycin, probably due to the antiproliferative effect of rapamycin. This finding might be relevant when interpreting TCS in RTRs.

**Supplementary Information:**

The online version contains supplementary material available at 10.1186/s12882-022-02741-7.

## Background

In the course of chronic kidney disease (CKD) and after transplantation as well, neurologic manifestations may develop [1, 2]. The underlying pathophysiology is likely to be multifactorial; however. inflammation contributes to the development of central nervous system injury [1]. Besides uremic encephalopathy [3], the most common neurologic complication, patients with end-stage renal disease (ESRD) may also develop movement disorders [3–5]. There is a growing body of evidence that idiopathic Parkinson’s disease (PD) may also occur in ESRD patients [6–9] and in patients with an estimated glomerular filtration rate less than 15 ml/min/1.73m^2^ [7]. The use of neuroimaging has increased in recent years in clinical studies on patients with PD [10, 11]. However, the considerable costs and the use of radioactive tracers are important caveats of implementation in routine clinical practice [12]. In recent decades, the use of transcranial sonography (TCS) of the cerebral parenchyma has gained wide acceptance as a diagnostic approach to movement disorders [13]. The finding of an enlarged substantia nigra (SN) echogenic area has been recognized as a sonographic marker of PD [14].

The pathophysiology of increased SN echogenicity is still not fully understood, but iron deposition seems to account for it [15]. It is well-known that, before successful renal transplantation, almost every patient has been kept on dialysis for years, eventually receives blood transfusion, and has been routinely given iron supplements and erythropoietin [16]. Therefore, it is plausible to assume that these patients meet underlying conditions for being iron overloaded [17, 18].

Inflammation is another relevant issue in renal transplant recipients (RTRs), mainly as a risk factor for cardiovascular events [19]. Besides, inflammation is also involved in atherosclerosis [20, 21], which in turn is thought to be also a risk for neurodegeneration [22, 23]. Two well-known sonographic parameters, the Doppler resistive index (RI) and carotid intima-media thickness (cIMT), are considered indirect markers of atherosclerosis [24–26].

With these premises in mind, the aim of the current study was to evaluate long-term RTRs to determine brain structure values by TCS and their relationship with derived Doppler RI of the middle cerebral artery, kidney graft and spleen, as well as cIMT.

## Methods

This was a single-center prospective observational survey in which long-term RTRs irrespective of renal function and healthy controls who agreed to participate in the study were selected. Between May 2018 and November 2020, consecutive RTRs referred for renal sonography were eligible for the study group, provided the following criteria were fulfilled: a) on triple-drug immunosuppression; b) without known neurological or psychiatric disease (not treated on an inpatient or outpatient basis); c) without known hepatobiliary disease and portal hypertension; d) aged at least 18 years; and e) with a transplant duration of at least 360 days. Control group participants were deemed to be eligible if they did not have proteinuria (urinary spot > 30 mg/dL) or increased serum creatinine (> 1.4 mg/dL) and denied having systemic arterial hypertension on treatment or diabetes mellitus. No neurological examination was carried out in any participant.

Data collection included demographic data (age, sex), underlying cause of renal failure, donor type, transplant duration, dialysis vintage, serum creatinine level, immunosuppressive regimen; transcranial sonographic measurement of mesencephalon and SN area, third ventricle width and middle cerebral artery (MCA) Doppler RI; abdominal sonographic measurement of spleen size, Doppler RI of native or graft kidney and spleen; sonographic measurement of cIMT.

All examinations of the brain, kidney, spleen and carotid were performed at the same run. Ultrasound Doppler examinations were performed using an Aplio 400 (Toshiba, Tokyo, Japan) equipment. A convex transducer (3.5 MHz) was used for native and graft kidney and spleen, a phased-array (2.0 MHz) for TCS and a linear probe (10.0-MHz) for cIMT. All TCS and carotid examinations were performed by two senior operators (RCLF and NCA) and all kidney graft and spleen Doppler ultrasound examinations were performed in the morning by the same operator (NCA) with the same routine.

### Transcranial sonography protocol (Figure S1)

A more extended description of the protocol has been provided in a previous publication [27]. Images were obtained bilaterally through the temporal bone window in axial planes. The bone window may not be permissive uni or bilaterally. TCS parameters studied were mesencephalic area and SN echogenic area, cerebral third ventricle width, and Doppler RI of the MCA at the M1 segment.

On the workstation of the equipment, the ipsilateral mesencephalon and the mesencephalic SN hyperechogenicity seen in both sides of brain were manually encircled. For each patient, only the largest SN and mesencephalic area was chosen for analysis, or, in the case of a unilateral permissive bone window, the only one available was chosen irrespective of the side. The width of the third ventricle was measured as the distance corresponding to the anechoic space between inner hyperechogenic lines formed due to significant acoustic impedance between ependymal walls and the cerebrospinal fluid.

The goal was to compare the TCS parameters values between both groups studied and their relationship with demographic data, Doppler RI, spleen size and cIMT. Additionally, a subset of RTRs were compared according to immunosuppressive regimen.

### Kidney/spleen ultrasound Doppler protocol (Figure S2)

A more detailed description of sampling has been already provided elsewhere for the kidney graft and spleen [28]. Briefly, the RI measurements were sampled at the level of the three interlobar arteries, in the upper, middle and lower poles of the kidney graft. The spleen was scanned in a manner to visualize the hilum. The RI of three arteries at the level immediately after perforating the spleen capsule was measured.

The RI was manually measured with built-in software and the average of the measurements in kidney and spleen was taken. Machine settings were adjusted for optimal performance. The Doppler angle was always zero.

### Carotid intima-media thickness (cIMT) measurement protocol (Figure S1)

The ultrasound exam to determine the cIMT of the left and right common carotid arteries was performed according to standardized protocols previously reported [25, 26]. Briefly, the right and left common carotid arteries were examined using a 10.0 MHz linear probe positioned anterolaterally in the cervical segment with the patient in the supine position. Using the built-in software of the ultrasound system, 1 to 2 cm proximal to the carotid bulb, the cIMT was measured from the intima-lumen interface until the media-adventitia interface. The mean cIMT of the two measurements was averaged to obtain the mean cIMT. Measurements including conspicuous plaques were avoided.

### Statistical analysis

Data were analyzed using SPSS software, version 17. Differences in demographics, TCS parameters and laboratory variables between renal transplant recipients and controls, were calculated using the Mann–Whitney U Test for continuous variables and chi-squared tests for categorical variables. Data analysis among different immunosuppressant regimens and controls was performed using the Kruskal–Wallis test, and Bonferroni post hoc tests were used to determine the differences between particular pairs of groups. Statistical relationships between variables were assessed using Pearson’s correlation coefficient. A *p*-value less than 0.05 was used as a cut-off for statistical significance.

The study was approved by the local ethics committee (CAAE: 57,851,816.6.0000.5259). All participants gave written informed consent.

## Results

TCS was performed in 184 participants, including RTRs (*n* = 156) and controls (*n* = 28). Out of 156 RTRs, 61 were excluded because of bilateral poor transtemporal insonation window (*n* = 15), short transplant duration time of less than 360 days (*n* = 34) or on only two-drug immunosuppression (*n* = 12). Among participants with normal renal function (28 controls) initially recruited for the study, one was excluded because of a poor window, three were ruled out because of hypertension, three because of proteinuria and one because of diabetes mellitus. After exclusions, 115 participants remained eligible for the study; 95 were RTRs (deceased donor, *n* = 59; living donor, *n* = 36). Immunosuppressant regimens included rapamycin (*n* = 22) or a calcineurin inhibitor (CNIs) (tacrolimus, *n* = 49; cyclosporine, *n* = 24) in addition to an antimetabolite (mycophenolate or azathioprine); in all instances, treatment regimens were administered in combination with steroids.

Twenty subjects with normal renal function, including renal donor candidates and employees among staff members, were assigned to the control group.

The main underlying etiologies (95 cases) of end-stage kidney disease were hypertension (*n* = 36; 36.8%), glomerulopathy (*n* = 20; 20.6%), diabetes mellitus (*n* = 6; 5.9%), polycystic kidney disease (*n* = 2; 2.9%), unknown causes (*n* = 21; 27.9%) and miscellaneous causes (*n* = 10; 5,9%). Patients with lupus erythematosus were assigned to the glomerulopathy group. None of the participants were on treatment for any neurologic disorder or had complaints of tremor.

Controls and RTRs had similar patterns of sex distribution (male, 50.0% vs. 57.9%; *p* = 0.517). Table 1 shows the continuous variables studied and the statistical differences between the controls and RTRs. RTRs were older, had higher creatinine levels, and larger SN echogenic area, third ventricle width and cIMT than controls. Doppler RI of the interlobar artery of the kidney graft of RTRs was higher than the renal interlobar artery of the kidneys of controls, although RI of the MCA and intraparenchymal spleen artery were similar.

**Table 1:** Comparison of demographic data, kidney, spleen and TCS parameters between controls and renal transplant recipients (RTRs)

Variable	Controls (*n* = 20)	RTRs (*n* = 95)	*P*
Sex, male	50.0%	57.9%	0.517
Age, y	43.05 ± 12.27	50.80 ± 13.64	**0.018**
BMI, kg/m^2^	25.92 ± 3.60	25.16 ± 4.61	0.170
Serum creatinine, mg/dL	0.83 ± 0.11	2.31 ± 1.55	** < 0.001**
Mesencephalon area, cm^2^	2.92 ± 0.35	2.76 ± 0.47	0.090
SN area, cm^2^	0.21 ± 0.07	0.30 ± 0.15	**0.012**
3^rd^ V, mm	2.62 ± 1.35	4.32 ± 2.06	** < 0.001**
cIMT, mm	0.55 ± 0.12	0.63 ± 0.15	**0.011**
MCA RI	0.52 ± 0.04	0.55 ± 0.09	0.288
Kidney RI	0.60 ± 0.06	0.71 ± 0.09	** < 0.001**
Spleen RI	0.54 ± 0.06	0.57 ± 0.08	0.277
Spleen size, mm	102.62 ± 11.72	104.24 ± 21.81	0.936

*TCS* Transcranial Sonography, *BMI* Body Mass Index, *SN* Substantia Nigra, *3*^*rd*^* V* Third Ventricle width, *cIMT* carotid Intima-Media Thickness, *MCA* Middle Cerebral Artery, *RI* Doppler Resistive Index

Patients treated with CNIs in comparison to patients on a rapamycin-based regimen had a shorter mean post-transplant time until the final period of data collection whereas dialysis vintage was similar in both groups. Renal function assessed by serum creatinine was the same in both patient groups and higher than in controls. The results showed that of all the ultrasound and Doppler parameters analyzed, the substantia nigra echogenic area was statistically larger in the CNIs group than in controls and the rapamycin group, while the third ventricle width was similar in both patient groups but larger than in controls. The spleen was statistically larger in the CNIs group than in the rapamycin group, but neither group was statistically different from control (Table 2). Between both immunosuppressant drug groups, there were no mean differences in renal, spleen or MCA Doppler RI. However, in the three territories assessed, Doppler RI of the graft kidney of both patient subsets was higher than in the native kidneys of controls, while no difference was found in the Doppler RI of the spleen or of MCA (Table 2).

**Table 2:** Comparison of data obtained from controls (*n* = 20), calcineurin inhibitors (CNIs, *n* = 73) and rapamycin (mTOR, *n* = 22) groups

Variable	Controls	CNI	mTOR	*P*
Age, years	43.05 ± 12.27	50.21 ± 14.26	52.77 ± 11.45	0.054
BMI, kg/m^2^	25.92 ± 3.60	25.11 ± 4.58	25.18 ± 4.84	0.389
Serum creatinine, mg/dL	0.83 ± 0.11	2.35 ± 1.66^a^	2.16 ± 1.14^a^	** < 0.001**
Transplant duration, days	-	3562 ± 2535^b^	5732 ± 3345	**0.004**
Dialysis vintage, mo	-	51.14 ± 48.26	53.91 ± 44.23	0.688
Mesencephalon area, cm^2^	2.92 ± 0.35	2.75 ± 0.45	2.80 ± 0.54	0.196
SN echogenic area, cm^2^	0.21 ± 0.07	0.31 ± 0.15^a, b^	0.23 ± 0.12	**0.002**
3^rd^ V, mm	2.62 ± 1.35	4.26 ± 2.07^a^	4.50 ± 2.05^a^	** < 0.001**
cIMT, mm	0.55 ± 0.12	0.63 ± 0.14	0.65 ± 0.17^a^	**0.040**
MCA RI	0.52 ± 0.04	0.55 ± 0.09	0.56 ± 0.09	0.539
Kidney RI	0.60 ± 0.06	0.71 ± 0.09^a^	0.71 ± 0.08^a^	** < 0.001**
Spleen RI	0.54 ± 0.06	0.57 ± 0.09	0.55 ± 0.07	0.355
Spleen size, mm	102.62 ± 11.72	107.36 ± 21.47^b^	93.87 ± 20.07	**0.026**

The mean differences among the three groups were assessed using the Kruskal–Wallis test, and Bonferroni post hoc tests were used to determine the differences between particular pairs of groups. The Mann–Whitney test was used in the case of only two groups

*BMI* Body Mass Index, *SN* Substantia Nigra, *3*^*rd*^* V* Third Ventricle width, *cIMT* carotid Intima-Media Thickness, *MCA* Middle Cerebral Artery, *RI* Doppler Resistive Index

^a^vs Controls

^b^vs Rapamycin

Correlation analysis to detect a relationship between increased SN echogenic area and third ventricle width with the independent variables are shown in Table 3. In controls, no relationship was found between SN and any of the studied variables, while the third ventricle showed a direct correlation with aging and cIMT. In RTRs as a whole group, SN area increased with spleen enlargement, while the third ventricle had a direct correlation with age, cIMT and Doppler RI of the MCA, graft kidney and spleen. In patients taking CNIs, the SN correlated positively with age and cIMT, while the third ventricle showed the same correlations seen in the whole group, i.e. it was positively correlated with age, cIMT and Doppler RI of the MCA, graft kidney and spleen. Rapamycin-based immunosuppressive regimen patients showed a direct relationship between the third ventricle and age and Doppler RI of the MCA, graft kidney and spleen; no statistically significant correlations were found with the SN.

**Table 3:** Correlations between TCS parameters (SN echogenic area and third ventricle width) and variables studied in controls and patient groups (RTRs – renal transplant patients; CNIs – calcineurin inhibitors; mTOR – rapamycin)

Variable	Controls		RTRs		CNIs		mTOR	
	SN	3rd V	SN	3rd V	SN	3rd V	SN	3rd V
Age								
r	-0.050	0.657	0.174	0.606	0.291	0.581	-0.303	0.722
p	0.833	**0.002**	0.092	**0.000**	**0.013**	**0.000**	0.170	**0.000**
Serum creatinine								
r	-0.085	0.441	0.001	-0.076	0.036	-0.083	0.360	0.016
p	0.828	0.235	0.991	0.537	0.798	0.553	0.206	0.958
Transplant duration								
r	-	-	0.088	0.083	0.223	0.139	0.046	-0.107
p	-	-	0.394	0.425	0.058	0.240	0.838	0.635
Mesencephalon area								
r	0.118	0.016	0.131	0.138	0.158	0.177	0.109	**0.017**
p	0.620	0.946	0.205	0.184	0.181	0.133	0.629	0.941
3^rd^ V								
r	-0.063	-	0.001	-	0.031	-	-0.063	-
p	0.791	-	0.989	-	0.796	-	0.781	-
cIMT								
r	-0.019	0.450	0.136	0.329	0.303	0.331	-0.368	0.317
p	0.937	**0.046**	0.189	**0.001**	**0.009**	**0.004**	0.092	0.150
MCA RI								
r	0.247	-0.224	-0.033	0.407	0.019	0.372	-0.229	0.528
p	0.308	0.356	0.755	**0.000**	0.879	**0.002**	0.331	**0.017**
Kidney RI								
r	0.084	0.150	**0.020**	0.418	0.053	0.356	-0.137	0.657
p	0.739	0.552	0.845	**0.000**	0.659	**0.002**	0.544	**0.001**
Spleen RI								
r	-0.345	0.130	-0.011	0.502	-0.054	0.484	0.067	0.648
p	0.148	0.595	0.920	**0.000**	0.652	**0.000**	0.773	**0.001**
Spleen size								
r	0.065	-0.375	0.291	-0.121	0.223	-0.087	0.347	-0.203
p	0.793	0.114	**0.004**	0.245	0.058	0.465	0.113	0.364

*SN* Substantia Nigra area, *3*^*rd*^* V* Third Ventricle width, *BMI* Body Mass Index, *MCA* Middle Cerebral Artery, *RI* Doppler Resistive Index, *cIMT* carotid Intima-Media Thickness

## Discussion

To the best of our knowledge, this is the first study to address SN echogenic signals using TCS in renal transplant recipients. The frequent occurrence of neurological complications in patients with CKD [1, 29] and RTRs [2] justifies the need for further investigations into this relevant issue. Indeed, kidney-brain cross-talk has attracted interest from researchers in the last decade [30–32].

From the clinical point of view, manifestations of patients with ESRD include frequent presentation of neurological disorders, such as cerebrovascular disease, cognitive impairment, and peripheral and autonomic neuropathies [1, 33]. Although in long-term dialysis patients the prevalence of stroke is as high as 17% compared to 4% for the general population [34] and global cognitive impairment occurs in 27% of these patients [35], a rate three times higher than that reported for age-matched general population [36], reports about the occurrence of PD and parkinsonism are limited to studies carried out in Asia [6–9].

The underlying pathophysiology of such neurological disorders is likely to be multifactorial, but the contribution of inflammation definitively plays a key role [1]. As far as one knows, RTRs satisfy criteria for a state of systemic inflammation [19]. Moreover, increased plasma chemokine levels, surrogate markers of inflammation, have been shown to be present even in clinically stable RTRs [37].

With this background, we investigated the SN echogenicity area and the cerebral third ventricle width of RTRs and controls. Among the obtained data, there are two sets of results that deserve comment, first regarding the differences in TCS parameters between controls and RTRs as a whole group and second in terms of the data reassessed after splitting the RTR group according to the immunosuppressive regimen. Accordingly, in this prospective study, the SN echogenic area and cerebral third ventricle width of RTRs were larger in comparison to controls.

One possible explanation for the underlying pathophysiology of increased SN echogenic area found in RTRs probably lies in inflammation. The inflammatory state associated with CKD increases the number of inflammatory cells in tissues of different organs, including the brain, known as gliosis [38]. In line with this topic, CKD in mice leads to reactive glial cells in brain regions including the SN [39]. With regards to sonography, this increased content of inflammatory cells in tissues is associated with increased echogenicity, as already demonstrated in the kidneys [40], in fat [41] and in tumors [42].

TCS is based on reflection and scattering of ultrasound waves at interfaces with different acoustic impedance. Displaying the echo pattern (echogenicity) of brain tissue TCS may provide new and complementary pathophysiological insights into disease states.

Different lines of evidence from experimental studies shed light on this topic. It is known that lipopolysaccharide injected into the rat SN evokes a typical inflammatory response that is followed by dopaminergic neurodegeneration [43], and an animal model of PD that results from injection of the neurotoxin MPTP (1-methyl-4-phenyl-1,2,3,6-tetrahydropyridine) involves an inflammatory process associated with microglia activation [44]. Therefore, it is reasonable to hypothesize that, in RTRs, the increased echogenicity of the SN might be associated with inflammation.

In hemodialysis patients, iron deposition in gray matter, including the SN, have been already reported [45]. In line with this, it has been claimed that increased iron content may be associated with increased echogenicity of the SN in PD patients [46]. Moreover, microglia might play a role in the iron status of their environment [47]. It is then reasonable to speculate that iron deposition in the SN secondary to cumulative dialysis before transplantation might have contributed to increased SN echogenicity found in the present study.

The increased cerebral third ventricle width found in our patients deserves to be highlighted, since it has been reported as a parameter for cerebral atrophy [48, 49]. Moreover, whole brain atrophy and ventricular expansion confirmed neurodegenerative disorders in autopsy proven diagnosis [50]. Therefore, this finding suggests that RTRs might have a higher degree of neurodegeneration in comparison to controls, although the extent of this brain damage did not produce overt neurological symptoms. Another possible explanation for the increased ventricular volumes of RTRs may rely in the fact that they were older than controls, merely representing the known enlargement of ventricular size that accompanies normal aging [51–53]. On the other hand, larger cIMT in RTRs than in controls provides further support to the hypothesis that RTRs are at increased risk for brain parenchymal loss, as it reflects more extensive atherogenesis in these patients [54]. It is worth mentioning that controls and RTRs were sex-matched and had similar BMI and mesencephalon area.

Arterial Doppler RI increases with age [55]. In the current study, although RTRs were statistically older than controls, RI of the spleen artery and MCA were similar in both groups, suggesting that the magnitude of the difference in age was not enough to affect the RI. On the other hand, RI was higher in the graft kidney of RTRs than in the native kidney of controls, a finding in accordance with renal transplant dysfunction [56] as can be inferred from unequivocally increased serum creatinine levels in RTRs.

Further subgroup analysis according to immunosuppressive regimen showed that although the rapamycin and control groups displayed a similar mean SN echogenic area, patients receiving CNIs had significantly larger SN echogenic area than those receiving rapamycin and controls, an unexpected result. This finding could not be assigned to age mismatch. Controls and rapamycin groups had the larger age mismatch and nonetheless there was no difference in SN area. On the other hand, despite CNI and rapamycin groups were matched for age, rapamycin group showed a smaller SN area. Moreover, the direct correlation between spleen size and SN echogenic area in RTRs, seen in this study, suggests that this finding may have similar underlying mechanisms. There is evidence supporting the role of inflammation in spleen enlargement commonly found in CKD [57]. This finding changed the study focus, as it was not designed to search for differences in SN echogenic area between different immunosuppressive regimens. However, the finding bears a good relationship with the overall subject of the research. In any case, increased SN area following treatment with CNIs but not with rapamycin was an unexpected finding that requires further experimental and clinical studies. This unexpected finding might be clinically relevant and should be taken into account when interpreting the results of transcranial sonography imaging in RTRs.

Considering that inflammation might be involved in the pathophysiology of the enlargement of SN in RTRs, one can speculate that the larger echogenic area of SN in patients receiving CNIs in comparison to rapamycin could be explained by differences in the mechanisms of action of each medication on cell proliferation. Indeed, in experimental models of PD, tacrolimus was unable to inhibit glial proliferation [58], while rapamycin reduced astrocyte activation and the inflammatory response [59]. In line with the latter study, rapamycin is well-known as a potent inhibitor of the proliferation of most hemopoietic and lymphoid cell lines [60]. The smaller spleen size in the rapamycin group of our study favors an anti-proliferative effect of rapamycin on microglia within the SN as well. The effect of rapamycin on spleen size reduction has been already reported after kidney transplant [61] and was reproduced in the present study. Furthermore, mTOR inhibitors significantly reduced tumor volume in tuberous sclerosis [62].

Although RI of the kidney and spleen and cIMT has been suggested to reflect the burden of cardiovascular factors and systemic atherosclerosis [26, 63, 64], and the association of inflammation with atherosclerosis is well-documented even in healthy populations [65], age, Doppler RI of the kidney, spleen and MCA as well as cIMT were not different between the CNIs and rapamycin groups. These data suggest that the larger SN area in CNIs group is not likely to be the consequence of more severe inflammation itself. However, the correlation analysis showed a clear relationship between SN area and risk factors for neurodegeneration like age and cIMT, but only in the CNIs group in contrast to the rapamycin group, suggesting that rapamycin was able to blunt the enlargement of the SN area associated with inflammation, likely due to its antiproliferative action. Further, both groups shared a demonstrable relationship between age and Doppler RI of the graft kidney and spleen and cerebral third ventricle width. Hence, it is reasonable to assume that rapamycin, in contrast to its effect on SN echogenic area, had no effect on third ventricle dilatation, a phenomenon reported in the normal ageing process [51–53].

As patients were free of neurologic complaints, a correlation could not be established between our imaging findings and neurological symptoms. Nonetheless, our results provide the first evidence that SN echogenic area is increased in renal allograft recipients taking CNIs, even though none of them actually have a history of PD.

It is tempting to also speculate that increased SN echogenic area might be related to the well-known neurotoxicity of CNIs, leading to some movement disorders [66], in a similar way that increased SN echogenicity is related to neuroleptic induced parkinsonism [67]. However, to further elucidate the underlying relationship between increased SN echogenic area and neurological side-effects of CNIs, longitudinal studies are necessary.

Although the present study results show an enlarged echogenic SN area in patients without complaints of tremor, any implication of this finding for the diagnostic approach of preclinical PD is beyond the scope of the current study.

One weakness of this study is that, even in the absence of neurological-related complaints, the participants should have been submitted to a thorough neurological evaluation in order to detect eventual mild symptoms not reported. The second weakness is that serum markers of inflammation were not included in the the study. However, cIMT can be used as surrogate marker of inflammation. Indeed, the association between cIMT and inflammatory markers like interleukin-6 [68, 69] and C-reactive protein [68, 70, 71] has been already reported.

## Conclusion

RTRs taking CNIs present a larger SN area than patients with a rapamycin-based immunosuppressive regimen.

## Supplementary Information

**Additional file 1. **Supplemental FigureS1 and Supplemental Figure S2**.** 

## Data Availability

The datasets used and/or analysed during the current study are available from the corresponding author on reasonable request. The requirement should first be submitted to ethical committee.

## Abbreviations

cIMT: Carotid intima-media thickness
CNIs: Calcineurin inhibitors
CKD: Chronic kidney disease
ESRD: End-stage renal disease
MCA: Middle cerebral artery
mTOR: Mammalian target of rapamycin
PD: Idiopathic Parkinson´s disease
RI: Doppler resistive index
RTRs: Renal transplant recipients
SN: Substantia nigra
TCS: Transcranial sonography
