# Supplementary material for: Transcranial sonography depicts a larger substantia nigra echogenic area in renal transplant patients on calcineurin inhibitors than on rapamycin
Source: BMC Nephrol. 2022 Mar 17;23:108. doi: 10.1186/s12882-022-02741-7 (PMC8931960; doi:10.1186/s12882-022-02741-7)
Supplement: Supplementary file 1 — Additional file 1. Supplemental FigureS1 and Supplemental Figure S2. [file 12882_2022_2741_MOESM1_ESM.pdf]

Figure S1

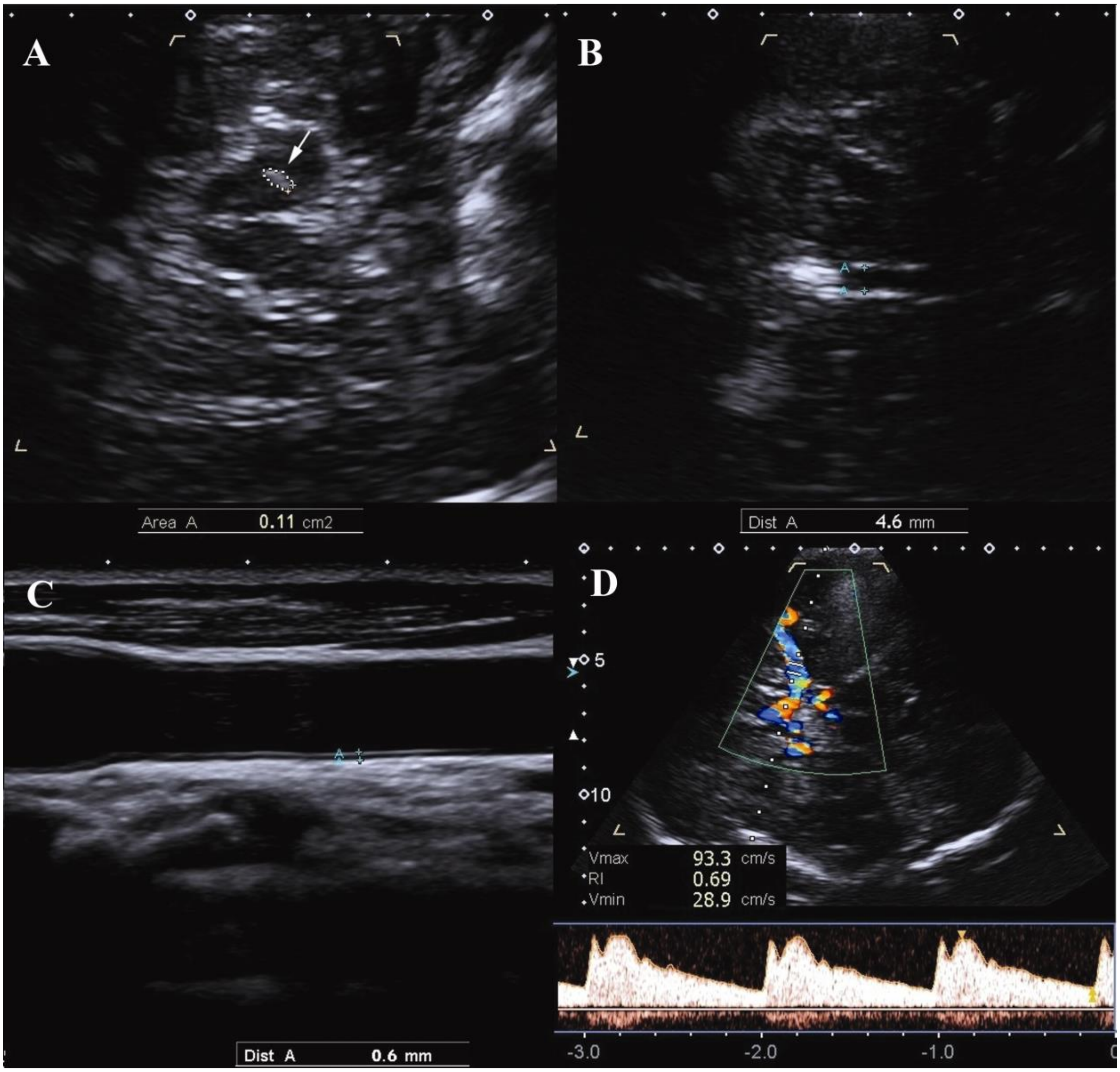

Figure S1 – (A) In the ipsilateral hypoechoic butterfly-shaped mesencephalic brainstem, the SN hyperechogenic area was manually encircled (arrow); (B) the cerebral third ventricle width was measured between the inner hyperechogenic lines of the ependymal walls (calipers); (C) 1 to 2 cm proximal to the carotid bulb, the cIMT was measured from the intima-lumen interface until the media-adventitia interface (calipers); (D) measurement of Doppler RI of the middle cerebral artery at the M1 segment.

Figure S2

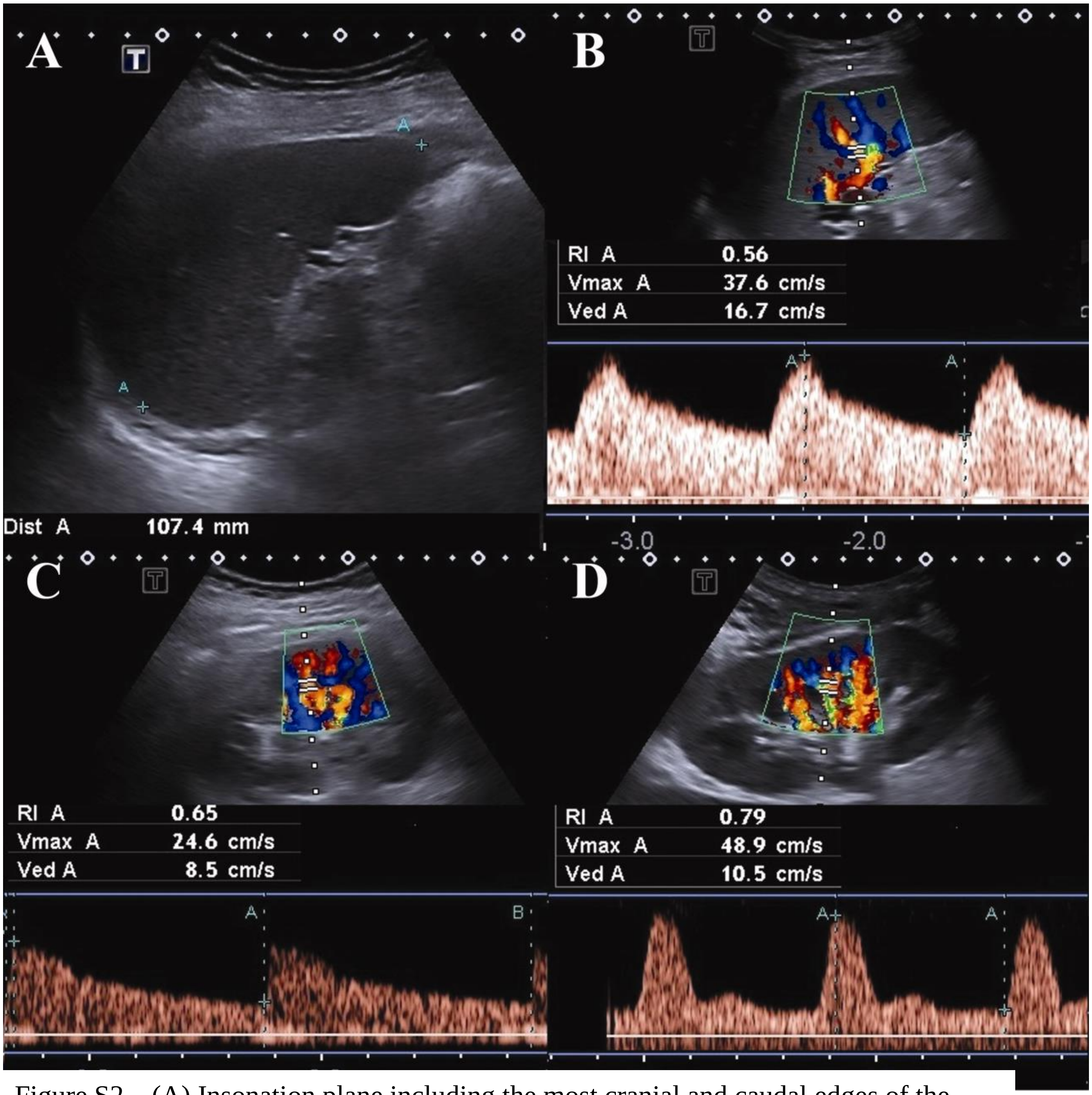

Figure S2 – (A) Insonation plane including the most cranial and caudal edges of the spleen for measuring spleen length (calipers); (B) intrasplenic artery Doppler RI at the level immediately after perforating the spleen capsule; (C) Doppler RI measurements were sampled at the level of the interlobar arteries of the native kidney; (D) Doppler RI measurements were sampled at the level of the interlobar arteries of the kidney graft.
